# Assessment of Knowledge and Utilization of Prefabricated Band and Loop Space Maintainers in Primary Dentition Among Dentists: A Cross-Sectional Questionnaire Study

**DOI:** 10.7759/cureus.65711

**Published:** 2024-07-29

**Authors:** Balaji Suresh, Ganesh Jeevanandan, Vignesh Ravindran

**Affiliations:** 1 Department of Pediatric and Preventive Dentistry, Saveetha Dental College and Hospitals, Saveetha Institute of Medical and Technical Sciences, Chennai, IND

**Keywords:** space loss, preventive orthodontics, band and loop, prefabricated space maintainer, space maintainer

## Abstract

Introduction: Preserving primary dentition is essential for maintaining optimal oral health and development in children. Premature loss of primary teeth due to caries, infection, crowding, or trauma can necessitate orthodontic intervention and lead to various complications. Space maintainers are critical for preserving arch space until the eruption of permanent teeth, thereby preventing issues such as ectopic eruption, crowding, and malocclusion. Despite their advantages, prefabricated space maintainers (PSMs) are underutilized. This study aims to evaluate the knowledge, attitudes, and practices of Indian dentists regarding PSMs in primary teeth.

Methodology: A cross-sectional questionnaire survey was conducted among 100 dental practitioners in Chennai. A 10-item self-administered questionnaire, developed based on a comprehensive literature review and expert consultations, assessed demographics, knowledge of PSM indications and techniques, current practices, perceived barriers, and preferences for continuing education. The questionnaire's reliability was confirmed with a Cronbach’s alpha value of 0.85. Descriptive statistics, including frequencies and percentages, were used to summarize the participants' demographic characteristics, knowledge levels, and current practices related to PSMs.

Results: Of the 100 respondents, 86 (86%) were males and 14 (14%) were females. Only 19 (19%) reported using PSMs, while 36 (36%) used conventional space maintainers. A significant proportion (42 (42%)) of the respondents held a master's degree in dental surgery, yet only 11 (11%) had participated in Continuing Dental Education (CDE) programs on space maintainers. The perceived benefit of PSMs being a single appointment procedure was acknowledged by 82 (82%) of the respondents, whereas 76 (76%) participants identified cost as a major drawback. Notably, 45 (45%) practitioners did not consider PSMs necessary.

Conclusion: This survey highlights notable obstacles in the adoption of PSMs among Indian dentists, emphasizing the need for focused educational initiatives. Improving knowledge and practices related to PSMs can enhance pediatric dental care and oral health outcomes in India.

## Introduction

The preservation of primary dentition plays a critical role in ensuring proper oral health and development in children [1]. It can be challenging due to premature loss of primary teeth resulting from dental caries, infection, crowding, or trauma, which may necessitate orthodontic treatment [2]. The treatment also has a variety of repercussions based on tooth loss, the child’s existing alignment, and occlusion [3]. In these cases, space maintainers work to keep the place of deciduous dentition within the arch until the permanent teeth emerge and occupy their proper position [3,4]. Although they are underutilized, space maintainers are crucial clinical interventions with numerous therapeutic and preventive advantages. They help in reducing the severity of negative outcomes such as ectopic eruption, crowding, and poor molar relationship and can be easily adapted for use without extensive customization [5].

Traditional band and loop space maintainers have been extensively used with a low failure rate, making them the most convenient method for space maintenance, as reported in previous literature [6]. In modern times, prefabricated band and loops are gaining popularity over conventional band and loop space maintainers as they can be delivered to the patient in the same appointment with no laboratory work and time expenditure at a comparably affordable cost, and have reported an 84.4% success rate [7]. The band selection of the prefabricated space maintainer is based on the mesiodistal width of the abutment tooth. Loop components are connected to the middle third of the band [8].

In India, oral health remains a significant public health concern due to various socio-economic factors and access disparities. So understanding the knowledge and practices of Indian dentists regarding prefabricated space maintainers (PSMs) becomes essential [9]. The utilization of PSMs can vary among dentists due to factors such as educational background, training received, clinical experience, patient acceptance, compliance, and financial factors [10]. While some dental practitioners may routinely incorporate PSMs into their treatment protocols, others may face challenges related to awareness, availability of resources, or concerns about efficacy [11]. The effectiveness of PSMs relies heavily on the dentist's awareness of their indications, correct placement techniques, and maintenance protocols [7]. However, there is a dearth of existing literature regarding dental practitioners' understanding and utilization of these devices. 

The objective of this questionnaire survey is to present the knowledge, attitudes, and practices of Indian dental clinicians regarding PSMs in deciduous dentition. By exploring these aspects, the study seeks to identify gaps in knowledge, barriers to utilization, and opportunities for improvement in dental education and clinical guidelines. Understanding these factors is crucial for enhancing the quality of pediatric dental care and promoting evidence-based practices among dental professionals.

## Materials and methods

Study design

Employing a cross-sectional questionnaire, the study investigates the knowledge and practices of dentists concerning PSMs in primary teeth. A questionnaire-based approach is chosen to gather quantitative data efficiently and comprehensively across a diverse sample of dental practitioners. The study was conducted over one month from June 1 to June 30, 2024. Participants provided informed consent, emphasizing confidentiality, voluntary participation, and the right to withdraw from the study at any time without repercussions.

A convenience sample of 100 dental practitioners in Chennai was included in this study. The target population included diverse participants from different practice types (e.g., private clinics, public hospitals, and academic institutions) and with varied experience levels. 

Questionnaire development

A 10-item self-administered questionnaire was developed in English (Table 1 in the Appendix) to assess the knowledge and utilization of PSMs in the primary dentition. The questionnaire design was based on an extensive review of the literature on PSMs and consultations with experts in pediatric dentistry [8,10,11]. It covered demographic variables (for example: age, gender, years of professional experience), understanding of PSM indications and techniques, current utilization patterns, perceived barriers to adoption, and preferences for continuing education on PSMs. The questionnaire was subjected to validation through repeated administration to a panel of five dentists over a one-week interval, yielding a Cronbach’s alpha coefficient of 0.85, signifying robust internal consistency. Respondents were instructed to choose the most suitable responses, thereby enhancing the reliability and validity of the survey instrument. The 10-question format ensures quality and completeness of responses while maintaining a high response rate among busy dental professionals.

Data collection

Data were collected electronically using online survey platforms to facilitate efficient distribution and collection of responses. Potential participants received an invitation to participate via online forums and direct email invitations to registered dentists.

Data analysis

Quantitative data collected from the survey responses were analyzed using appropriate statistical methods. The collected data was entered into a Microsoft Excel (2019 version 2406; Microsoft, Redmond, WA, USA) spreadsheet and subjected to statistical analysis. All analyses were conducted using the IBM SPSS Statistics, version 26 (IBM Corp., Armonk, NY, USA). Descriptive statistics, including frequencies and percentages were used to summarize the participants' demographic characteristics, knowledge levels, and current practices related to PSMs.

## Results

**Figure 1:**
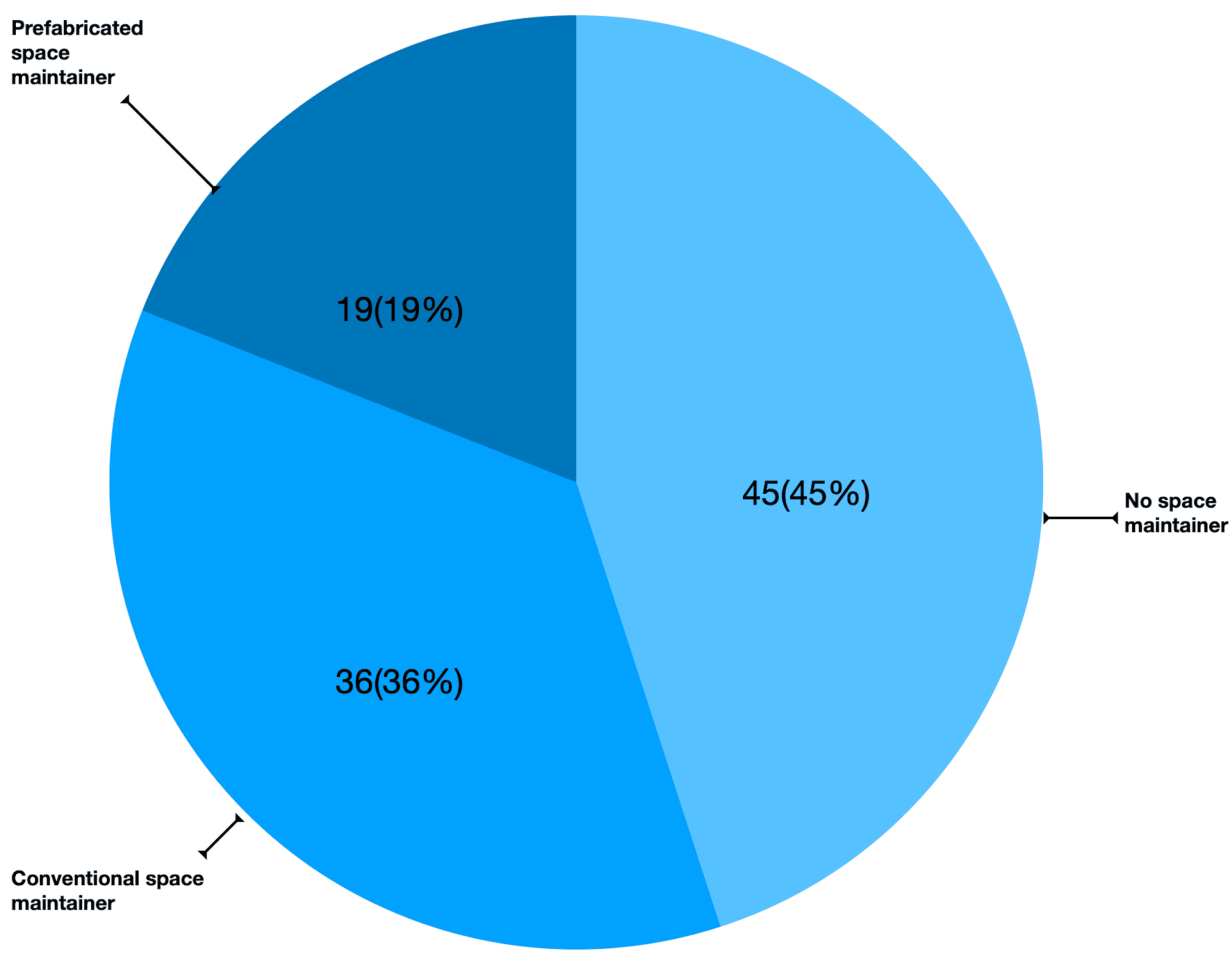
Distribution of space maintainer types in dental practice

In the current study, 100 practitioners participated, comprising 86 (86%) males and 14 (14%) females. The findings indicate that 19 (19%) participants utilize PSMs, while 36 (36%) practitioners employed conventional space maintainers for primary teeth (Figure 1).

**Figure 2:**
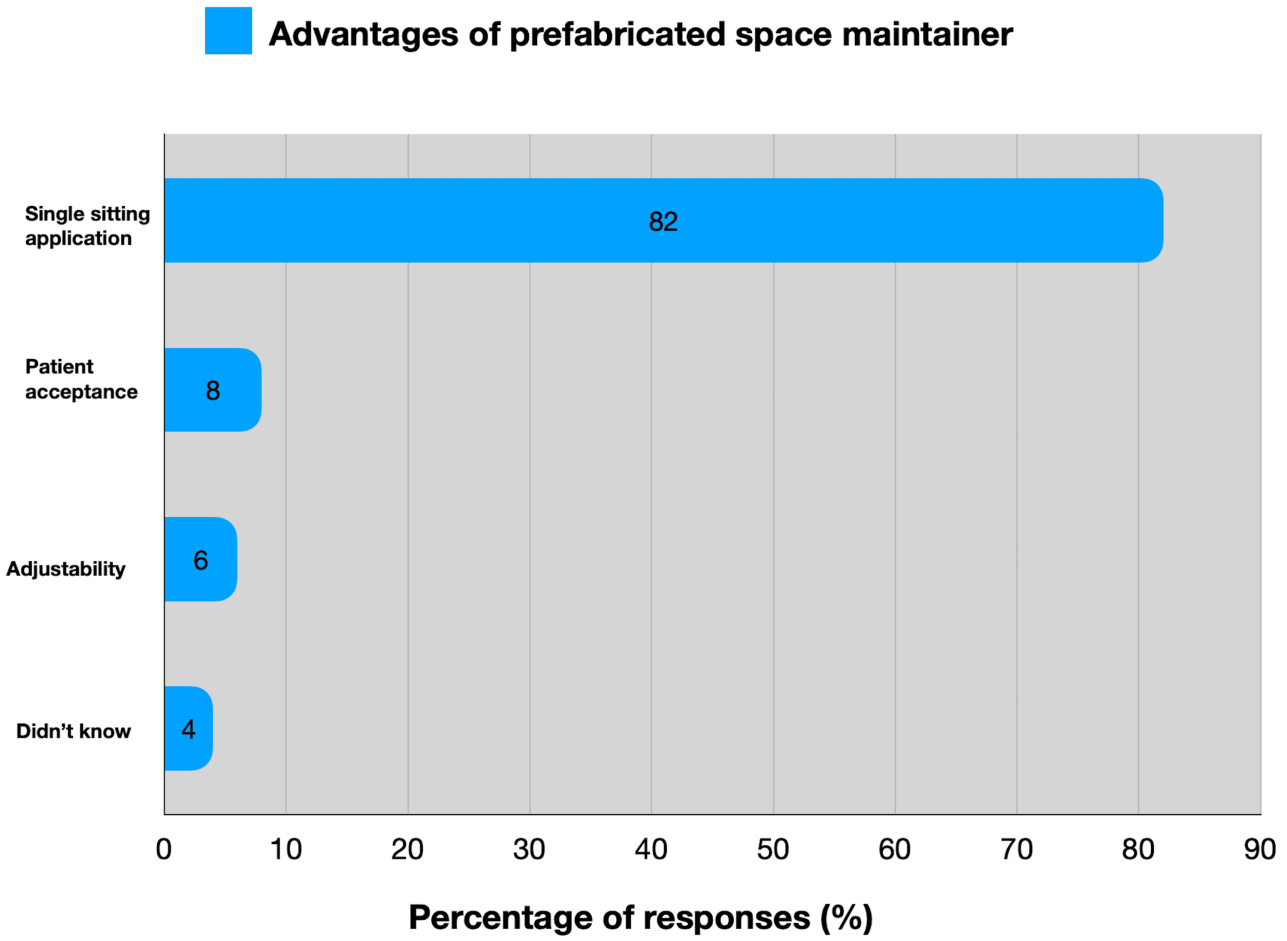
Distribution of the perceived advantages of using prefabricated space maintainers

Among the participants, 42 (42%) held a master's degree in dental surgery, and only 11 (11%) had attended Continuing Dental Education (CDE) programs specific to space maintainers. Regarding the use of PSMs, 82 (82%) of the respondents identified the single-sitting appointment as the primary advantage over traditional options (Figure 2).

**Figure 3:**
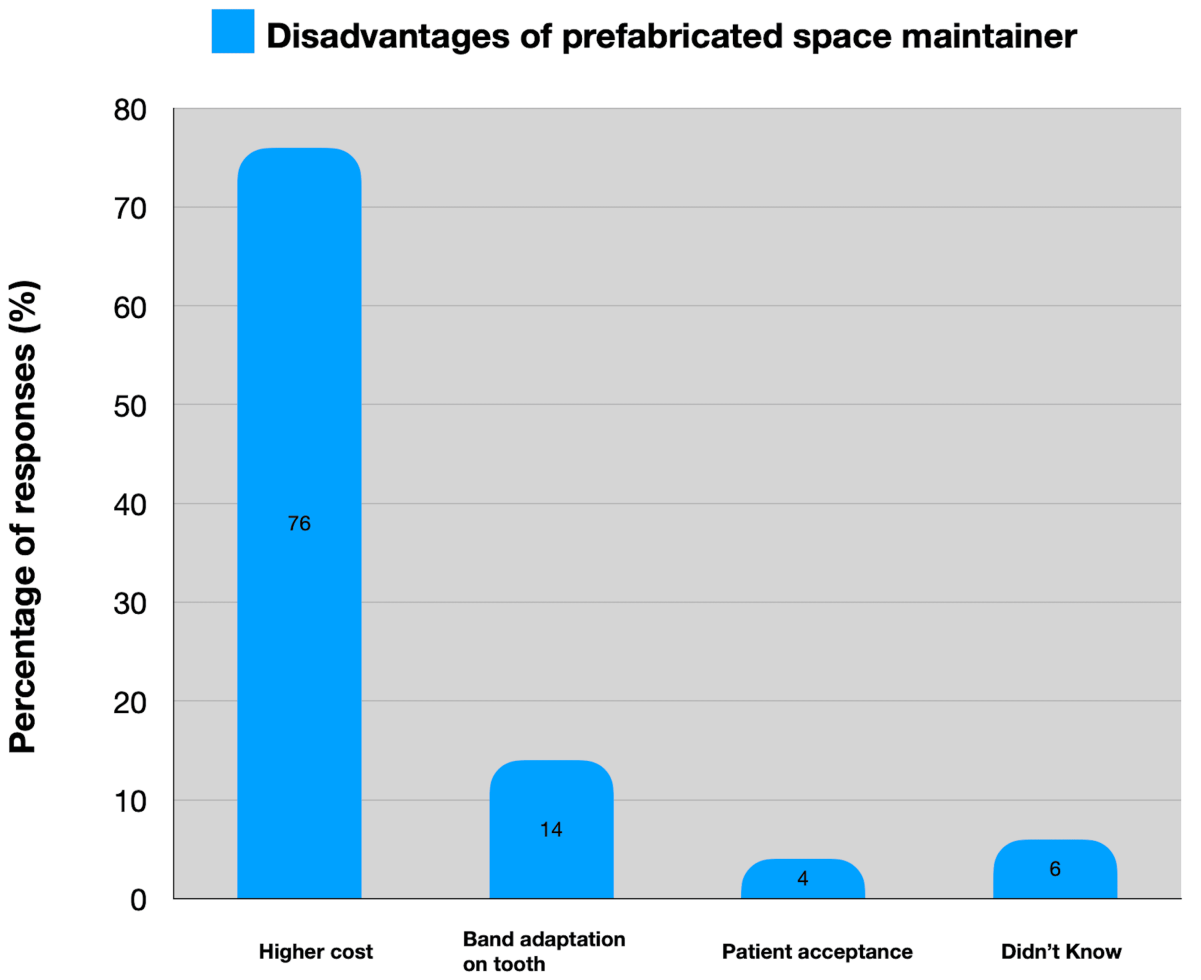
Distribution of the perceived disadvantages of using prefabricated space maintainers

Conversely, 76 (76%) respondents cited higher expenses as the major drawback of PSMs (Figure 3).

**Figure 4:**
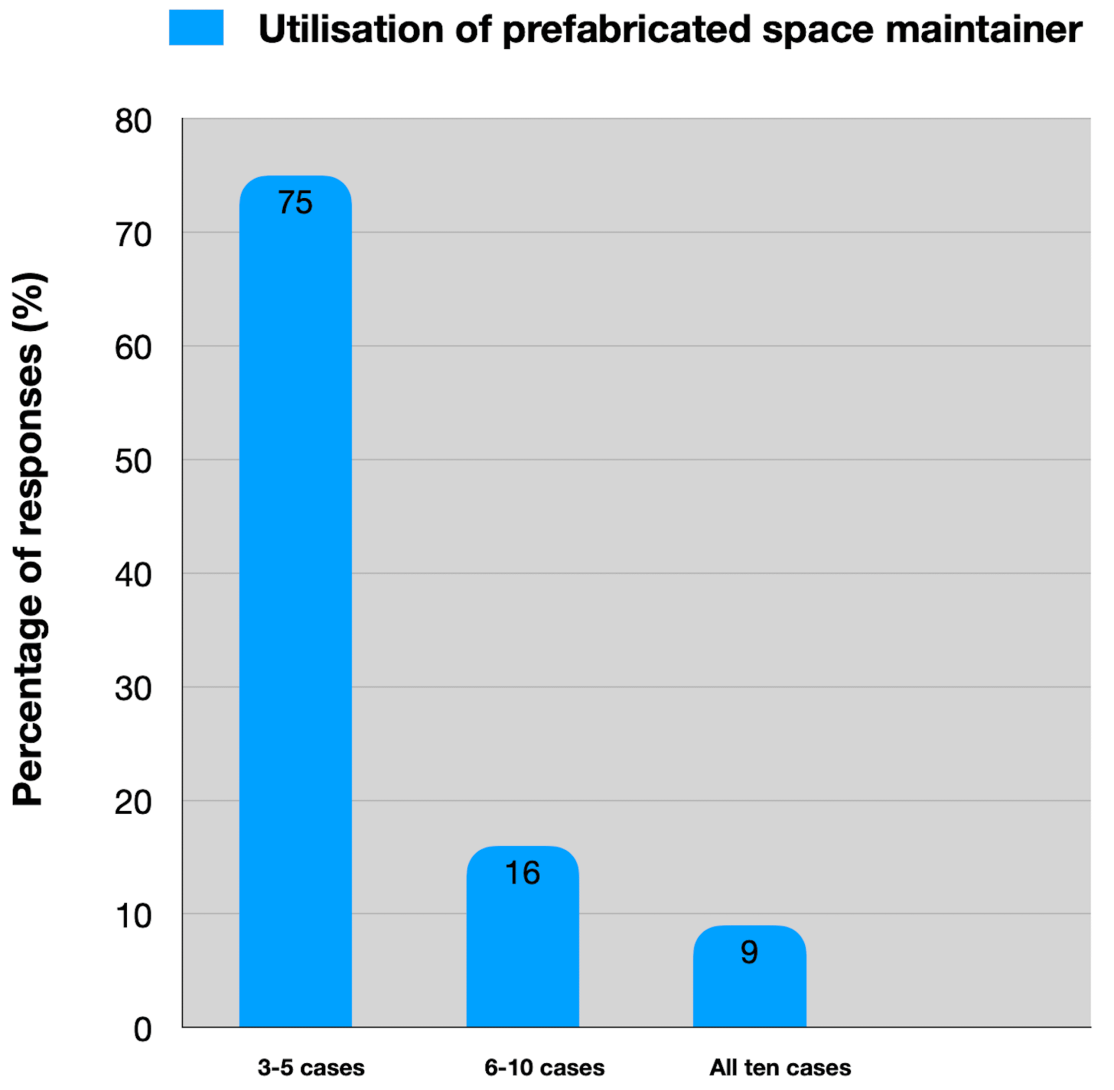
Distribution of the perceived utilization of prefabricated space maintainers

Both arches have the same level of accessibility with PSMs. Among the practitioners who used PSMs, 75 (75%) of them utilized it for three to five cases, 16 (16%) used them for six to 10 cases, and 9 (9%) used them for all the 10 cases. PSM was not deemed necessary by 45 (45%) of the practitioners (Figure 4).

## Discussion

This research provides substantial insights into the contemporary practices and perceptions among dental professionals regarding space maintainers, specifically focusing on prefabricated options versus conventional methods. Additionally, the study explores their educational backgrounds and utilization patterns. Firstly, the demographic breakdown of the practitioners shows a significant gender disparity, with 86 (86%) being male and 14 (14%) being female. This gender distribution might influence perspectives and practices within the field, reflecting broader trends in dental demographics.

Regarding the use of space maintainers, the study highlights that only 19 (19%) practitioners utilize PSMs, while 36 (36%) opt for conventional space maintainers for primary teeth. This indicates a preference for traditional methods despite the benefits associated with prefabricated options.

Educational background is another crucial aspect discussed in the findings. A notable number (42 (42%)) of practitioners hold a master's degree in dental surgery, suggesting a higher level of specialization among the study participants. However, only 11 (11%) have attended Continuing Dental Education (CDE) programs focused on space maintainers. This gap in CDE attendance underscores a potential opportunity for enhancing knowledge and adoption of newer technologies and techniques, such as PSMs.

The study also explores practitioners' perceptions of PSMs. Conventional band and loop space maintainers have several limitations, such as needing a minimum of two appointments and being difficult for children who are uncooperative or have gag reflexes as they require impression models for fabrication of the appliance. These are in addition to a long list of flaws and disadvantages, such as cement dissolution, soldering failure, decay on the sideline band, and time-consuming construction [12,13].

Multiple scholarly articles have underscored that the conventional band and loop method stand out as the most practical approach for maintaining dental space, noted for its extensive utilization and proven effectiveness in clinical settings. Prefabricated bands and loops have been introduced to dentistry in recent years and simply need one appointment, fit into a session fast, don't require laboratory work, take less time, and are reasonably priced [14,15]. In the present study, the convenience of a single-sitting appointment is highlighted as a significant benefit by 82 (82%) practitioners, illustrating the practical advantages that appeal to practitioners seeking efficiency in their clinical practice. Conversely, higher expenses are identified as the primary drawback by 76 (76%) respondents, indicating a barrier to wider adoption despite perceived benefits.

In the study by Setia et al., prefabricated bands and loops exhibited superior gingival health outcomes (72.8% within nine months) compared to conventional types (45.7% within nine months), indicating greater gingival compatibility and potentially lower inflammation in the gingiva [16]. Additionally, our findings highlight comparable accessibility between arches using PSMs, suggesting their practical utility in various dental scenarios for managing space maintenance. However, it is important to note that prefabricated band and loop space maintainers do not guarantee the success of the soldered joint, which is a significant disadvantage of PSMs.

Usage patterns among PSM users reveal that 75% utilize PSMs for three to five cases, with smaller percentages using them for higher case numbers. This distribution suggests that while PSMs are integrated into practice, they may not yet be extensively employed across all cases that could benefit from their use. According to Abdulhameed et al.'s study, loop fracture and cement dissolution accounted for 86.6% of the cases with broken bands and loops over a 12-month period [17]. In a 20-month study, the success rate of traditional bands and loops was 40%, with 82% of the failures being attributable to solvent cement, according to a study by Sasa et al. [18].

A striking finding is that nearly 45 (45%) practitioners do not consider PSMs necessary, indicating varying opinions on the efficacy and applicability of these devices within the dental community. The study's contribution is to advance knowledge in the field of pediatric dentistry. It underscores how the findings fill gaps in current understanding and provide a foundation for further research and improvement in clinical practice. It emphasizes the importance of space maintainers in preventing dental problems early in life and underscores the role of preventive care in overall oral health [19,20]. This proactive approach can reduce the burden of dental disease and associated healthcare costs over the long term. The limitations of this study include the potential for response bias arising from participants' self-reported data on their knowledge and practices. Additionally, the use of convenience sampling may constrain the generalizability of the study's findings to the broader population of Indian dentists. However, efforts will be made to mitigate these limitations through a careful questionnaire design, pilot testing, and transparent reporting of the study methods and findings.

## Conclusions

This study underscores the importance of PSMs in maintaining space in primary dentition. Despite their documented efficiency and potential cost-effectiveness, their utilization among dentists remains suboptimal. The findings reveal notable knowledge gaps and inconsistent practices, with a prevailing preference for conventional methods over PSMs. These gaps highlight the need for targeted educational initiatives and Continuing Dental Education programs to enhance the understanding and acceptance of PSMs. Addressing barriers such as cost perceptions and necessity through evidence-based training could facilitate greater adoption of PSMs. Enhanced integration of PSMs into clinical practice is likely to improve the management of primary dentition and overall oral health outcomes for children in India. Further research is recommended to explore the long-term efficacy and practical challenges associated with PSMs.

## Appendices

**Table 1:** Questionnaire for evaluating knowledge and utilization of prefabricated space maintainers in primary dentition CDE: Continuing Dental Education

S. No	Question	Choose the best
1	Do you use a prefabricated band and loop space maintainer as a space maintainer in primary teeth?	Yes/No
2	Have you attended any workshop or CDE program regarding prefabricated space maintainers in primary teeth?	Yes/No
3	Which space maintainer system you use in primary teeth?	Conventional /Prefabricated /None
4	What is the greatest advantage of using prefabricated space maintainer primary teeth compared to conventional space maintainer?	Single-sitting application/ Patient acceptance/ Adjustability/ I don't know
5	What is the greatest disadvantage of using prefabricated space maintainer in primary teeth?	Higher cost/ Band adaptation on tooth/ Patient acceptance /I don't know
6	Ease of access using prefabricated space maintainer primary tooth is more in?	Maxillary arch/ Mandibular arch /Same in both /I don't know
7	What is the disadvantage in the use of conventional space maintainer in primary teeth?	Requires multi-visit /Requires impression/ Patient acceptance/ I don't know
8	How often do you use prefabricated space maintainer in pediatric case? (out of ten cases)	1-2/ 3-5/ 6-10/ All
9	How often do you use conventional space maintainer in pediatric case? (out of ten cases)	1-2/ 3-5/ 6-10/ All
10	Do you think there is a necessity for a prefabricated space maintainer in primary teeth?	Yes/No
